# Social vulnerability and its possible relation to the principal causes of morbidity and mortality in the Mexican state of Oaxaca

**DOI:** 10.1186/s12939-018-0849-2

**Published:** 2018-09-03

**Authors:** Ana María González-Villoria, Roberto Ariel Abeldaño Zuñiga

**Affiliations:** Guillermo Rojas Mijangos S/N, Miahuatlán de Porfirio Díaz, Universidad de la Sierra Sur, Oaxaca, Mexico

**Keywords:** Environmental health risk, Indoor air pollution, Unsafe water

## Abstract

The health status of a population is the conjunction of many biological, political and social factors. The biological representations of diseases are attributed to factors, such as ischemic heart disease, that are attributed to unhealthy lifestyles when individuals have high levels of cholesterol and triglycerides, or lack of physical activity, or to genetic factors while ignoring social factors such as poverty. This study observes how morbidity and mortality of the population could be affected by living conditions.

## Introduction

Vulnerability is defined as the susceptibility to be harmed, or as the degree to which a system is susceptible and cannot withstand adverse effects [1]. It is marked by different factors: biological, cultural, social, economic, political, geographic, as well as diseases, educational level, age, gender, social status, behaviour and housing, which make certain groups more sensitive to premature and excessive morbidity and mortality. Moreover, these factors are not mutually exclusive [2]. Consequently, the same groups can present these vulnerabilities or disadvantage factors in a cumulative way; hence, those groups that tend to accumulate a greater number of factors are more likely to suffer injury or have a specific priority need in comparison to the rest of the population [2].

The state of Oaxaca, like every state within the Mexican Republic, has access to priority health programs; however, it ranks among the last places in health indicators in the national health surveys. Why does this state present such disparity? A possible answer could be found by analysing different social and economic factors related to vulnerability in order to clarify the main issues that may affect this phenomenon, so that the major health needs of the people of Oaxaca would be considered.

## Methodology

To carry out the social study of living conditions we analysed reports of the National Institute of Statistics and Geography (INEGI) [3], Ministry of Health (SESA) [4] and the National Survey of Health and Nutrition (ENSANUT 2012) [5], as well as citizen reports of access to health care in Oaxaca. Once the social data were obtained, health conditions were compared between the states ranked from 1 to 32, with the lowest and highest degrees of social vulnerability [6] The ranking of the principal factors associated with diabetes and hypertension, living conditions and principal causes of mortality were obtained by dividing the rank occupied by the state by the total number of states (32) Mexico has 32 states, so the highest value implies the worst conditions. World Health Organization (WHO) [7] reports and published scientific articles were consulted for biological studies associated with social conditions. Finally, the work data was analysed for morbidity and mortality related to living conditions in Oaxaca.

### Health status of the Oaxaca population

The state of Oaxaca is located in the southeast of Mexico. It is divided into eight regions: the Isthmus, Mixteca, Sierra Sur, the Coast, Sierra Norte, Central Valleys, Tuxtepec or Papaloapan, and Cañada; furthermore, it is composed of 570 municipalities. The population in 2015 was 3,967,889, 2,079,211 women and 1,888,678 men [3].

In 2013, life expectancy was 72.5 years, which is two years less than the national average; not only is life expectancy lower, a healthy life expectancy is 63.6 years in men and 67.7 years in women, which implies that at least five years of life will be unhealthy [4].

The principal diseases vary by age; the National Survey of Health and Nutrition 2012 (ENSANUT by its initials in Spanish) states that the main causes of childhood morbidity are acute respiratory infections (ARI), followed by diarrheal diseases and urinary tract infections (UTI) [5]. ARIs are presented as the most prevalent in adolescents but the prevalence is modified, as UTIs are listed in second place and diarrheal disease in third. Finally, coupled with these conditions, adulthood hypertension is reported to appear from the age of 20 and diabetes from 40, as shown in Table 1.

**Table 1:** Health status of Oaxaca

	Morbidity			Mortality		
	Women	Men	Total	Women	Men	Risk Factors commonly associated to diseases
1	Acute respiratory infections	Acute respiratory infections	Acute respiratory infections	Diabetes	Diabetes	Hyperglycaemia
2	Urinary tract infections	Intestinal infections by other organisms and some poorly defined	Intestinal infections by other organisms and some poorly defined	Ischemic heart	Ischemic heart	Bad dietary habits
3	Intestinal infections by other organisms and some poorly defined	Urinary tract infections	Urinary tract infections	Cerebrovascular diseases	Cirrhosis	Overweight and obesity
4	Gastric ulcers and duodenitis	Conjunctivitis	Gastric ulcers and duodenitis	Hypertensive disease	Homicides	Consumption of alcohol and drugs
5	Acute vulvovaginitis	Gastric ulcers and duodaenitis	Conjunctivitis	Malnutrition	Cerebrovascular diseases	Arterial hypertension
6	Conjunctivitis	Acute otitis media	Acute otitis media	Chronic respiratory diseases	Hypertensive disease	Low glomerular filtration
7	Acute otitis media	Gingivitis and periodontal diseases	Gingivitis and periodontal diseases	Cirrhosis	Traffic accidents	Malnutrition
8	Gingivitis and periodontal diseases	Intestinal amoebiasis	Acute vulvovaginitis	Acute respiratory infections	Chronic respiratory diseases	High cholesterol
9	Intestinal Amoebiasis	Febrile syndrome	Intestinal Amoebiasis	Renal insufficiency	Acute respiratory infections	Low physical activity
10	Febrile syndrome	Pharyngitis, streptococcal tonsillitis	Febrile syndrome	Congenital	Malnutrition	Smoking

Data from the Informe de Salud de los Mexicanos 2015. Principle causes of morbidity and mortality in Oaxacans

Ten of the previously mentioned diseases are responsible for years of lost healthy life in adults. Diabetes is the number one cause, followed by chronic kidney disease. These conditions highlight the evident economic impact, that despite having acceptable levels of education it has been observed that in different regions work absenteeism caused by illness increases vulnerability [6].

### Mortality

The first three causes of Oaxacan mortality noted in Table 1 are shared with the rest of the country, diabetes being the most significant, followed by ischemic and cerebrovascular diseases with a slightly different variation according to sex.

Amid the main causes of Oaxacan morbidity and mortality are some diseases where the state is at a significant disadvantage compared to others, such as Mexico City or Nuevo León, that have the lowest indexes of social vulnerability. For example, Oaxaca occupies different places in male and female mortality due to malnutrition. Malnutrition is the fifth leading cause of death in women and tenth in men, nevertheless, mortality due to malnutrition is nearly non-existent in Mexico City and Nuevo León [8].

The principal illnesses in the states of Mexico with the highest and lowest social vulnerability reported by the Ministry of Health are displayed in Table 2. According to the level of health, the state of Oaxaca, compared to other states with lower vulnerability indexes, has the highest possible value (32) and, therefore, a lower degree of health.

**Table 2:**
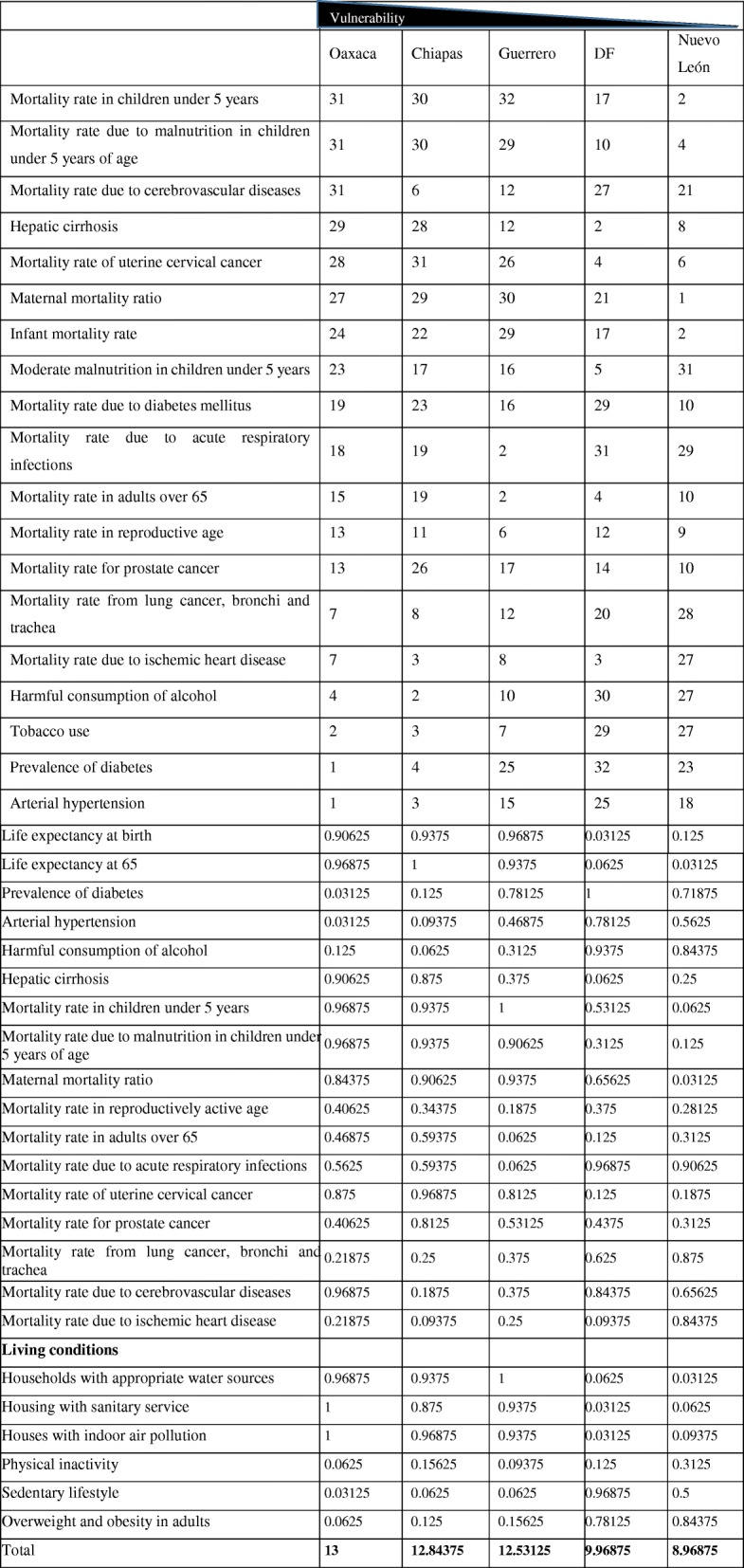
Illnesses in the states of the Mexican Republic with the highest and lowest social vulnerability

Data from the Informe de Salud de los Mexicanos 2015 [4] The table shows the ranking of the principal illnesses in the states with the most and least index of vulnerability. The highest value represents the worst conditions.

The table shows the ranking of the principal factors associated with diabetes and hypertension, living conditions and principal causes of mortality [4].

Among the risk factors associated with mortality in the state are: hyperglycaemia, poor dietary habits, overweight and obesity, alcohol and drug consumption, hypertension, low glomerular filtration rate, malnutrition, high cholesterol, low physical activity and smoking [4].

### Social factors health related

Although mortality associated factors are biological, it is worth considering other factors that are present in the everyday environment, particularly poverty. 65.9% of the population is impoverished, far above the national rate of 49.5%, and indicators such as education, housing and access to health services are far below national averages [4].

A high percentage of Oaxacans speak an indigenous language (34%). The state’s unique characteristic is that most of the population is distributed around the 245 municipalities where 26 indigenous languages are spoken, and one- fifth of the inhabitants do not speak Spanish. The predominant languages are Zapotec, Mixtec, Mazatec and Chinantec [9].

### Education

Despite having great cultural diversity, the educational level is very low. 6% of the population is illiterate, more than twice the national level (13%); the average educational level of persons older than 15 is 7.5 years of formal schooling, lower than the national level of 9.2; 11% have never attended school, and finally, 51.8% of the population has a low educational level [3].

### Housing

It is important to highlight that 65.9% of the population live in conditions of poverty, a situation that reflects the lack of basic services; it is also important to note that more than 20% of the population are without access to appropriate water sources, 31.2% are without access to sanitary services (disposal of excreta), 11% of the houses have dirt floors-an index markedly above the 3.2% national average- only 43.2% of the houses are built with a roof of resistant material and 11.6% of homes do not have water, electricity or gas, therefore occupying the penultimate place in the nation [4, 10]. Furthermore, these conditions of poverty have a direct impact on health conditions, particularly water and indoor air pollution [11, 12].

According to the World Health Organization (WHO) there are several diseases related to water caused by faecal-oral transmission of microorganisms and chemical substances present in drinking water [7]. Diseases such as ascariasis, onchocerciasis, typhoid, schistosomiasis, campylobacter infection, cholera, diarrhoea and paratyphoid enteric fevers; these diseases are related to faecal-oral transmission by non-potable water. Parasitic diseases arise from a contaminated water source, which produces conditions such as diarrhoea that in conjunction with collateral risk factors generate various problems. The probability of parasitic illness is increased by consuming water from unsuitable groundwater tables or by drinking water that includes an intermediate mollusc, causing water-borne diseases such as schistosomiasis [7, 11, 13–16].

However there are other diseases that have to do with the lack of access to water, such as malaria, where the transmission and reproduction of the *Aedes aegypti* mosquito is related to water storage. Once the water has been collected, it must be stored, which increases the possibility of disease-bearing vectors, such as malaria, Zika virus and Chinkungunya, transmitted by *Aedes aegypti* [15–18] Villagers have a long walk to get clean water, and as a result, the same sanitary issues that concerned people in the early nineteenth century are still current in Oaxaca.

A fundamental feature of theses living conditions is that 49.5% of homes are saturated with polluted air, ranked as the last place in the country [4]. This polluted air is generated by the use of solid fuels such as wood, agricultural waste, charcoal, coal and animal excrement for cooking and heating inside the home. [12]

These fuels produce large amounts of particles such as soot, which penetrates deep into the lungs [19, 20] moreover, if in addition homes are poorly ventilated with dirt floors, there is an increase of air pollutant particles, resulting in even more unfavourable living conditions.

Health issues arise from such exposure, and women and children are the most affected. It is well-known that being exposed to indoor air pollution almost doubles the risk of pneumonia during childhood and that more than half the deaths in children less than five years of age are caused by acute lower respiratory infection due to inhalation of indoor air contaminated by solid fuel [12].

It is well known that particle-polluted air produces respiratory diseases, such as cancer, asthma and emphysema, and drastically increases the probability of suffering acute respiratory infections. In fact, WHO has estimated that one out of every three respiratory infections worldwid, is associated with exposure to polluted air [12]. The results of inhaling particles generated by combustion, depending on the specific particle, are irritation, inflammation and hyper-reactivity; these particles reduce mucociliary and thus, macrophage response, they are also haemoglobin bound and thus reduce oxygen transport; the more bronchial reactivity, the more susceptibility to infections, even carcinogenic ones [21]. The damage caused by this pollution is considered to be as alarming as tobacco smoke [22].

While the respiratory effects of contaminated air inhalation is well known, WHO reports that of the 4.3 million people in the world who die prematurely due to inhaling polluted air, 12% is due to respiratory diseases, specifically pneumonia, while the rest are due to other conditions that are probably not related to that exposure; 34% cerebrovascular accidents, 26% ischemic heart disease, 22% chronic obstructive neuropathy, and 6% due to lung cancer. Contaminated air inhalation has even been associated with diabetes and preeclampsia [23]. It is important to highlight that such conditions include three of the main causes of morbidity and mortality in Oaxaca.

Once pollutants enter the body through the respiratory tract producing chronic inflammation, pro-inflammatory cytokines are secreted, which triggers chronic inflammation and immunological responses. Once settled in the bloodstream these particles cause cardiovascular damage in various ways, such as the induction of oxidative stress, systemic inflammation, endothelial dysfunction, thrombosis that generates heart failure or ischemic diseases and arrhythmias. Additionally, accumulation of these contaminants in the arteries may form obstructions similar to those caused by high-fat diets [24].

A large number of diseases prevalent in Oaxaca are associated with living conditions as shown in Table 3, such as limited access to suitable water sources, lack of access to sanitary services, a proper place for excreta disposal or exposure to polluted air.

**Table 3:** Diseases prevalent in Oaxaca associated with vulnerability factors

Vulnerability factors	Place among the health risk*	Percentage	Associated diseases according to WHO
Access to suitable sources of water	Penultimate (31)	80%	Ascariasis (helminthiasis), malaria, onchocerciasis, schistosomiasis, campylobacteriosis, diarrhoea typhoid, paratyphoid enteric fevers, anaemia, malnutrition.
Houses with access to sanitary services	Last (32)	66%	
Indoor polluted air exposure	Last (32)	49.3%	Pneumonia, chronic obstructive pneumonia stroke, ischemic heart disease.
Indoor usage of solid fuel	Last (32)	49.3%	

Source: data from Informe de la Salud de los Mexicanos 2015 [4]

Summary of the 20 leading causes of disease in the state of Oaxaca that, according to WHO, are related to the lack of appropriate water sources, lack of access to sanitary services and the use of solid fuel in the home.

## Discussion

Urinary tract infections are associated with kidney failure and ischemic heart disease and cerebrovascular accidents are associated with high cholesterol, high triglycerides or obesity (64.6% of the population) and arterial hypertension. Ischemic heart disease, in the case of Oaxaca, cannot be attributed solely to the presence of high levels of serum carbohydrates, in view of the high level of physical activity of the population. With 90% of the population active, Oaxaca is positioned as the state with less physical inactivity, shown by the few hours that people spend in front of the television. This is probably a function of poverty, as people have to walk everywhere, cannot afford television, and live in areas without access to television or Internet signals.

Hence, it is necessary to consider that breathing contaminated air inside the home is a social factor that may be associated with the three main causes of mortality in Oaxaca, especially in women, since they are the most exposed to polluted air due to cooking and time spent within the home. When biological factors are not enough to explain causality, the answer is surely related to social factors.

To reduce morbidity and mortality in Oaxaca, in addition to complying with the vaccination programs and recommendations issued by WHO, it is necessary to analyse the possibility of environmental interventions that are closely related to health.

In 2015 the United Nations proposed that with the implementation of 17 global goals, life could be improved for people around the world. To achieve these objectives the united efforts of government, business and civil society is necessary. The goal of Sustainable Development Goals 3 (SDG3) is to ensure healthy lives and promote well-being for all. Additionally, there are health related SDGs, such as SDG6, that focus on ensuring availability and sustainable management of water and sanitation for all, and SDG 7, dedicated to ensure access to affordable, reliable, sustainable and modern energy for all [25]. SDG6 is directly related to SDG3, as providing good quality water reduces tropical diseases like malaria and water-borne diseases. The SDG reports mention that in Mexico, mortality attributed to household air pollution is 15.2%, but in Oaxaca it is much greater, at 23.5%, and unsafe water, unsafe sanitation and lack of hygiene is 1.1 per 100,000 population [25]. Although worldwide access to water sources and waste disposal has improved, the situation of different regions or states of a country may be far from the national averages [26] and local problems may be underestimated.

The lack of access to appropriate sources of water and the use of fuels other than gas or electricity are not problems that poor people can solve on their own in order to prevent the deterioration of their health. In some cases the solution may be unknown, their living situation places them at a disadvantage and they lack the resources to modify their environment.

The reduction of use of solid fuel for heating and cooking, and improved sanitation, is not a key objective in some countries because it is not directly related to non-communicable diseases. As Krieger points out, the disadvantages in social epidemiology are not mutually exclusive, [27] and in this case they are associated with the prevailing conditions in Oaxaca. Oaxaca displays a biological expression of social inequality, since people are incorporating pollutant particles into their bodies by inhaling them during their daily activities and thus, the population expresses biologically the experiences of economic inequality due to the impoverished conditions in which they live, leading to an untimely death [27]. The difference in health conditions with the rest of the country is not a matter of race or ethnicity, but of the living conditions of these ethnic groups. Women and children being the most affected, biological affectation has a lifelong trajectory development, which could be considered as “biological programming” in which the harm that has occurred during childhood has repercussions throughout the rest of life.

Although there has been an increase in health expenditures in the state of Oaxaca, it is quite obvious that there is still a lot more to be done. Despite the fact that a large part of the population has government health insurance (Seguro Popular) 15.9% do not have access to health services, and 83.8% do not have access to social security [4, 10]. Moreover, within the population that has access to health services, ENSANUT reports that in 2012 the rate of these services was 66.8%, lower than the 77% national rate [5] . Nevertheless, being affiliated with the health system does not necessarily indicate that access to the services is full and effective. Additionally, user reports on quality and effective access to health services in Oaxaca indicated that the people who were attended either did not receive their full medication prescriptions or had to pay for laboratory studies or medications during surgical treatment [28]. The actions of the SDGs related to health can reduce the main causes of morbidity and mortality in Oaxaca and places Mexico as one of the countries that improves the life of the population through the application and fulfilment of the SDG goals.

## Conclusions

As can be deduced, opening more clinics is not the solution, as there will probably be no doctors, not enough medicines and the location will be inaccessible to the population. If Oaxacans have the same rights to health services as the rest of the country, then the causes of social disadvantage are the immediate problem and the causes of morbidity and mortality which do not present risk factors such as lack of physical activity and/or diabetes, will require multifaceted action for health improvement, specifically in the construction of healthy environmental conditions.

Therefore, if actions were to be implemented to reduce the two factors of contaminated air and water that have been established as global objectives, as in the millennium development goals of SDGs 3, 6 and 7, Oaxaca’s morbidity and mortality would be reduced. WHO has suggested a plan called Water, Sanitation and Hygiene, with the objective of accelerating and sustaining progress in respect to unattended tropical diseases (WASH) and thus to control and eliminate them [29]. An excellent example is improving health by clearing the air to reduce premature mortality from non-communicable diseases. Policies implemented to reduce the exposure to dangerous particles have reduced cardiovascular mortality from 17.8% in 2000 to 10.3% in 2015 [30].

Additionally it has been observed that changing fuel material, ventilating homes or increasing the quantity of multivitamin tablets, increases the function of the immune system and thus reduces respiratory infections. These measures have been taken by other countries to reduce the production of pollutants within the home [31, 32]. And, as with any measure, it must go hand in hand with national health programs.

## Abbreviations

ARI: Acute respiratory infection
ENSANUT: National survey of health and nutrition
INEGI: National Institute of Statistics and Geography
SESA: Ministry of health
UTI: Urinary tract infection
WHO: World Health Organization
